# Better Organization in Medicine

**Published:** 1895-01

**Authors:** L. D. Hill

**Affiliations:** Webberville, Texas


### Correspondence.

### Better Organization in Medicine.

WEBBERVILLE, December 8, 1894.

*Editor Texas Medical Journal:*

You ask for suggestions as to the best means of strengthening and perpetuating permanently the Texas State Medical Association. This is an important question, and one in which every lover of his noble profession ought to be interested.

There are several impediments to be overcome before the Association can be what it should be,—the representative of every regular physician in Texas. The prerequisites for success, are permanency of location, and a permanent membership. The first could be attained by *locating* the headquarters of the Association either at Austin or Galveston, where the Association through its officers would be brought in touch with the departments at Austin and the Medical School at Galveston; thereby giving their combined support to the Medical School and Health Officer of the State, and in return receive their aid and influence in securing proper legislation to protect the people, and the Medical School of the State. This would meet the first prerequisite for success, viz: permanency of location. The second can never be met by the annual *change* of headquarters that only secures the attendance of those nearest the place of meeting and who will never be heard of again, with a few exceptions. The membership must be as *permanent* as the location, to secure success.

How can this be accomplished? My plan would be to make the *State* Association a *representative* law making body, and the final court of appeals for all the medical organizations of the State. Give the county, district, and all other medical organizations now in the State one representative for every —— members of said organization, to be *elected annually* by the society to which he belongs, and make every member of these minor organizations a member of and contributor to the State Association, by the payment of —— dollars per annum to the State Association, to be collected by the treasurer of the Society of which he is now a member, as a condition of membership and good standing in either society. Charge $5.00 for each new member joining any medical organization in the State, $1.00 to go to the State Association for every new member. It is estimated that there are 4,000 regular physicians in the State; at one dollar per capita that would give $4,000 for current expenses, then add one dollar for each new member, and I think you would have no trouble to run the State Association on a firm and permanent financial basis. With a permanent organization as indicated, I think the State could be induced to furnish domicile for the State Medical Association and its archives. Then the State Association would be a grand success. Every regular physician entitled to a seat at small cost with the certainty of a sufficient number of elected delegates present to transact the business of the Association. There are other organizations in the State with large membership, many of whom were never in the State organization who have a right to a seat when present, yet all pay tribute willingly to the State organization.

I send you these suggestions for what they are worth as the means of calling out the opinions of others.

Yours, L. D. HILL, M. D.

Webberville, Texas.

